# Supplementary material for: A true choice of place of birth? Swiss women’s access to birth hospitals and birth centers
Source: PLoS One. 2022 Jul 6;17(7):e0270834. doi: 10.1371/journal.pone.0270834 (PMC9258807; doi:10.1371/journal.pone.0270834)
Supplement: Attachment — Submitted filename: Responses to reviewers III.docx [file pone.0270834.s001.docx]

Basel, May 2022

Manuscript ID: PONE-D-21-14065

Dear Dr. Fischer,

The authors immensely thank the two reviewers for their supportive comments and suggestions to improve the manuscript. We have sought to incorporate the suggestions into the revised manuscript and are certain that the reviewers would find this new manuscript much improved and informative.

Please note that changes within the main document are highlighted in red.

Please also note that maps 1-4 were created by the authors themselves (and not obtained from a third-party) and thus are not copyrighted. Therefore, these maps can be published in their current form without any permission or consent from a possible copyright holder (see first comment below).

Sincerely,

The authors

| **Reviewer Comments** | **Author’s Response** | **page** |
| --- | --- | --- |
| **Additional PLOS ONE requirements to be addressed by the authors** | | |
| 1. We note your response: “"Please note that maps 1-4 were created by the authors themselves (and not obtained from a third-party) and thus are not copyrighted. Therefore, these maps can be published in their current form without any permission or consent from a possible copyright holder (see first comment in responses to reviewers). "  We also note that the data used to create the map was obtained from the Swiss Federal Statistical Office, however, where were the map images themselves obtained from? | The map images were created solely based on free geo-data. That means, there is no underlying image (that has been used). The map images result solely from calculations using the free geo-data provided by Geofabrik GmbH.  Data source:   - Geofabrik (<https://download.geofabrik.de/> ). On the website it says:   *„Welcome to Geofabrik's free download server. This server has data extracts from the* [*OpenStreetMap project*](http://www.openstreetmap.org/) *which are normally updated every day. Select your continent and then your country of interest from the list below. (If you have been directed to this page from elsewhere and are not familiar with OpenStreetMap, we highly recommend that you read up on OSM before you use the data.) This open data download service is offered free of charge by Geofabrik GmbH.”* | - |
| a) Where did the authors obtain the maps in Figures 1-4?  b) Please state whether the map images have been previously copyrighted to your knowledge.  c) If any of the map images have been previously copyrighted, we require specific consent from the copyright holder to publish these images in PLOS ONE, under the CC BY 4.0 license. | Please note that maps 1-4 were created by the authors themselves (and not obtained from a third-party) and thus are not copyrighted.  Therefore, these maps can be published in their current form without any permission or consent from a possible copyright holder.  Moreover, we have cited the underlying data sources used in our analysis in the manuscript. However, if accepted, we could add the following clarification to the maps (which we had not added to blind the manuscript).   | - |
| 1. Please ensure that your manuscript meets PLOS ONE's style requirements, including those for file naming. The PLOS ONE style templates can be found at <https://journals.plos.org/plosone/s/file?id=wjVg/PLOSOne_formatting_sample_main_body.pdf> and  <https://journals.plos.org/plosone/s/file?id=ba62/PLOSOne_formatting_sample_title_authors_affiliations.pdf> | The authors made sure the manuscript meets the style requirements. | 1-18 |
| 2. In your Data Availability statement, you have not specified where the minimal data set underlying the results described in your manuscript can be found. PLOS defines a study's minimal data set as the underlying data used to reach the conclusions drawn in the manuscript and any additional data required to replicate the reported study findings in their entirety. All PLOS journals require that the minimal data set be made fully available. For more information about our data policy, please see <http://journals.plos.org/plosone/s/data-availability>.  Upon re-submitting your revised manuscript, please upload your study’s minimal underlying data set as either Supporting Information files or to a stable, public repository and include the relevant URLs, DOIs, or accession numbers within your revised cover letter. For a list of acceptable repositories, please see <http://journals.plos.org/plosone/s/data-availability#loc-recommended-repositories>. Any potentially identifying patient information must be fully anonymized.  Important: If there are ethical or legal restrictions to sharing your data publicly, please explain these restrictions in detail. Please see our guidelines for more information on what we consider unacceptable restrictions to publicly sharing data: <http://journals.plos.org/plosone/s/data-availability#loc-unacceptable-data-access-restrictions>. Note that it is not acceptable for the authors to be the sole named individuals responsible for ensuring data access. | As pointed out in our data availability statement, we cannot make the study’s minimal data set publicly available, because parts of the employed data (i.e. highly disaggregated population data) were used under license (paid by the University of Basel). That means there are legal restrictions to sharing our data both publicly and privately. We changed our data availability statement. It reads as follows.  *The present study employed data from various sources. While highly disaggregated population data were used under license (University of Basel) for the current study and are not publicly available, all other data were open source. Population data are only available from the Swiss Federal Statistical Office. Thus, the dataset generated and analyzed during the current study can neither be made publicly available nor shared with others, since it employed the above mentioned population data. That means there are legal restrictions to sharing our data both publicly and privately. However, further results for smaller administrative areas (e.g. communities, cities) or comparisons between different geographical areas can be made available by the corresponding author upon reasonable request.*  *Moreover, interested readers can contact the Swiss Federal Statistical Office and order the respective populations statistics (*[*https://www.bfs.admin.ch/bfs/en/home/statistics/population/surveys/statpop.html*](https://www.bfs.admin.ch/bfs/en/home/statistics/population/surveys/statpop.html)*) which then can be combined with the other three data sources that are open source (see methods section), thereby creating the data set generated and analyzed during the current study.* | - |
| 3. We note that you have indicated that data from this study are available upon request. PLOS only allows data to be available upon request if there are legal or ethical restrictions on sharing data publicly. For more information on unacceptable data access restrictions, please see <http://journals.plos.org/plosone/s/data-availability#loc-unacceptable-data-access-restrictions>.  In your revised cover letter, please address the following prompts:  a) If there are ethical or legal restrictions on sharing a de-identified data set, please explain them in detail (e.g., data contain potentially sensitive information, data are owned by a third-party organization, etc.) and who has imposed them (e.g., an ethics committee). Please also provide contact information for a data access committee, ethics committee, or other institutional body to which data requests may be sent.  b) If there are no restrictions, please upload the minimal anonymized data set necessary to replicate your study findings as either Supporting Information files or to a stable, public repository and provide us with the relevant URLs, DOIs, or accession numbers. For a list of acceptable repositories, please see <http://journals.plos.org/plosone/s/data-availability#loc-recommended-repositories>. | We have addressed this issue in our response to the previous comment.  Also, please note that our first data availability statement indicated that “further results for smaller administrative areas (e.g. communities, cities) or comparisons between different geographical areas can be made available by the corresponding author upon reasonable request”, that is travel times to the two birth settings.  We did not indicate that data can be made available. | - |
| 4. Please include a caption for figures Map 1-4. | Figure captions are provided in the main document (see pages 8 & 10).  Captions:  Map 1. Geographical areas with true choice of birth setting  Map 2. Accessibility of birth hospitals  Map 3. Accessibility of birth centers  Map 4. Difference between travel times  Figure 1. Mean travel times for both birth settings | 8/10 |
| 5. Please ensure that you refer to Figures map 1-4 in your text as, if accepted, production will need this reference to link the reader to the figure. | Figures and maps are referred to in the text. | 8/10 |
| 6. We note that Figures Map 1-4 in your submission contain map images which may be copyrighted. All PLOS content is published under the Creative Commons Attribution License (CC BY 4.0), which means that the manuscript, images, and Supporting Information files will be freely available online, and any third party is permitted to access, download, copy, distribute, and use these materials in any way, even commercially, with proper attribution. For these reasons, we cannot publish previously copyrighted maps or satellite images created using proprietary data, such as Google software (Google Maps, Street View, and Earth). For more information, see our copyright guidelines: <http://journals.plos.org/plosone/s/licenses-and-copyright>.  We require you to either (1) present written permission from the copyright holder to publish these figures specifically under the CC BY 4.0 license, or (2) remove the figures from your submission. | Map 1-4 are not copyrighted, but were created by the first author (i.e. Sebastian Rauch). Hence, these maps can be published in its current form.  Also, if accepted, please make sure to make the original maps available (i.e. approx. 62MB/map), since in the compiled pdf they are of low quality.  The original maps allow zooming in until one can see each of the 254.278 Swiss 100x100 meters raster cells (in which women of childbearing age reside) and, thereby, provide important (“visualized”) data at a community or city level. | - |
| **Reviewer 1** | | |
| Thanks for allowing me to review your interesting manuscript. The results on accessibility are very interesting and I suspect could be replicated in many other countries. Given the relatively larger number of birth hospitals when compared to birth centres the difference in travel times is not that surprising.  However, choice of place of birth is dependent on many more things other than accessibility based on travelling times. For example, many birth centres (in my own experience) are limited in capacity and will only accept a limited number of bookings each month. Sometimes this is based on the models of care and the number of women each midwife or group of midwives can care for.  Do you have any data on capacity of each birth centre and to what extent this may also limit choices? | The authors agree that choice of place of birth is dependent on many factors. While birth centers are limited in capacity in Switzerland, there are no robust data available on limited capacities.  However, it is known that in response to the Covid-19 pandemic many families have reassessed their birth plans and awareness of and interest in out-of-hospital births (e.g. home birth, birth centers) have increased. Thus, it can be assumed that existing birth centers are even less sufficient (compared to pre-pandemic times) to care for all women who want to give birth in birth centers. We have expanded on this issue on page 13 (results and discussion section). It reads as follows:  *In light of birth centers’ limited capacities and an increased interest in out-of-hospital births, existing birth centers might be even less sufficient (compared to pre-pandemic times) to care for all women who want to give birth in a birth center. This is unfortunate, since increased access to birth centers has been recommended as a response to the challenges faced by pregnant women during the Covid-19 pandemic [51].* | 13 |
| **Reviewer 2** | | |
| The topic of the article is very interesting, since it is a topic to be discussed. I support to accept this article because it offers new perspective and novelty of the topic. I would recommend to accept to publish this article. | The authors thank the reviewer for the supportive comment. | - |
